# Two-Stage Cervical and Thoracic-Lumbar-Sacral Circumferential Fixation for Pyogenic Spondylitis: A Case Report

**DOI:** 10.7759/cureus.53070

**Published:** 2024-01-27

**Authors:** Yoshinori Maki, Kenji Fukaya

**Affiliations:** 1 Neurosurgery, Hikone Chuo Hospital, Hikone, JPN; 2 Rehabilitation, Hikari Hospital, Otsu, JPN; 3 Neurosurgery, Ayabe Renaiss Hospital, Ayabe, JPN

**Keywords:** percutaneous pedicle screw, oblique lumbar intervertebral fixation, posterior cervical fixation, anterior cervical discectomy and fusion, pyogenic spondylitis

## Abstract

Pyogenic spondylitis is a rare life-threatening condition. Conservative treatment with antibiotics is indicated; however, surgery can be considered in refractory cases. The surgical strategy varies, as pyogenic spondylosis can occur from the cervical to sacral regions. To our knowledge, although there is less invasiveness as an advantage in the following management, cervical and thoracic-lumbar-sacral circumferential fixations in two sessions for pyogenic spondylitis have not been previously described. An 84-year-old man complained of ambulation disturbances and pain in the neck and upper and lower extremities (the Japan Orthopaedic Association cervical myelopathy evaluation questionnaire score of 5/17). Magnetic resonance imaging revealed pyogenic spondylitis of the cervical, thoracic, and lumbar regions. Epidural abscesses and spondylodiscitis were concurrently diagnosed with multi-level skipping lesions from the cervical to the sacral regions. As these lesions were resistant to antibiotic treatment and the neurological symptoms worsened, surgical treatment was planned. Anterior cervical discectomy and fusion, and posterior cervical fixation were followed by oblique and posterior lumbar intervertebral fusions with long-level fixation from T12 to the ilium using percutaneous pedicle screws. The surgeries were performed in two sessions to avoid the invasiveness of surgeries in a single session. The patient’s condition improved after a second surgery. The patient was discharged on postoperative day 116. No recurrence was observed for six months, and the patient was able to ambulate independently. Two-stage cervical and thoracic-lumbar-sacral circumferential fixation for pyogenic spondylitis contributed to a favorable outcome (the Japan Orthopaedic Association cervical myelopathy evaluation questionnaire score of 13/17).

## Introduction

Pyogenic spondylitis is a rare infectious disease with an estimated annual of 0.4-2.0 per 100,000 cases [1,2]. It can be diagnosed concurrently with epidural abscess, discitis, and spondylodiscitis [3,4]. Although the thoracic and lumbar regions are relatively common locations for pyogenic spondylitis, it can occur anywhere from the cervical to sacral regions [5].

Conservative treatments including immobilization and antibiotics can be effective if pyogenic spondylitis is in the early phase [6]. However, surgical treatment is indicated in cases of refractory pyogenic spondylitis resulting in complications such as abscess formation, severe persistent pain, spinal instability and/or deformity, or neurological deficits due to spinal cord compression [3,4]. Surgical treatment is usually aimed at debriding infectious sites, and various surgical strategies are indicated according to the site of infection. Anterior, posterior, or combined anterior and posterior open surgery is typically indicated for pyogenic spondylitis [7]. In addition to these conventional approaches, following technological advancements, minimally invasive surgery via the posterior approach using percutaneous pedicle screws (PPSs) has been reported to be safe and effective for pyogenic spondylitis and spondylodiscitis [8,9]. The effectiveness of oblique lumbar intervertebral fixation (OLIF) for thoracic and lumbar pyogenic spondylodiscitis has also been described [10,11]. In previous studies, this novel approach has been performed at only a single or limited level [9-11]. To the best of our knowledge, a case with multilevel pyogenic spondylitis undergoing OLIF combined with PPSs from T12 to the ilium has not been previously described. Moreover, cervical circumferential fixation performed for a patient undergoing thoracic-lumbar-sacral circumferential fixation for pyogenic spondylitis has not been reported.

Here, we report the first successful management of pyogenic spondylitis spreading from the cervical to the sacral regions using cervical circumferential fixation, OLIF, and posterior lumbar intervertebral fixation (PLIF) combined with PPSs from T12 to the ilium performed in two sessions.

## Case presentation

An 84-year-old man presented for a scheduled follow-up by an otorhinolaryngologist. The patient was taking medications for hypertension, hyperlipidemia, and hypothyroidism. He complained of malaise, pain in the back and bilateral upper extremities, and numbness in the right and left upper extremities. These symptoms gradually worsened four days prior to admission. A blood sample examination showed evidence of inflammation: white blood cell and C-reactive protein levels were 8840/μL and 32.15 mg/dL, respectively. Thoracic and abdominal computed tomography revealed left-sided pleurisy. The patient was emergently admitted and tazobactam and piperacillin hydrate (4.5 g 3 times/ day) were transvenously initiated. After three days, a follow-up blood sample examination showed residual inflammation (white blood cell: 14250/μL, and C-reactive protein: 22.45 mg/dl). The pain in the upper bilateral and lower right extremities persisted. Motor weakness in the bilateral upper and lower extremities was also observed (manual muscle testing grade of 1-2 in the upper extremities, and of 2 in the lower extremities, respectively). The Japan Orthopaedic Association cervical myelopathy evaluation questionnaire score was 5/17. Magnetic resonance imaging (MRI) was performed to exclude a cervical spinal lesion and it revealed a pharyngeal abscess and cervical spondylosis. Initially, we believed that the patient’s neurological symptoms resulted from cervical spondylosis. Methicillin-susceptible Staphylococcus aureus was identified in the blood and urine cultures collected on admission. Therefore, antibiotic treatment was continued. However, three days later, the neurological symptoms aggravated, and the patient underwent whole-spine MRI. Edematous changes were observed in the vertebral bodies of C5 and C6, along with a collection of fluid in the intervertebral space of C5/6. Epidural fluid was observed from the cervical to the sacral vertebral levels.

**Figure 1:**
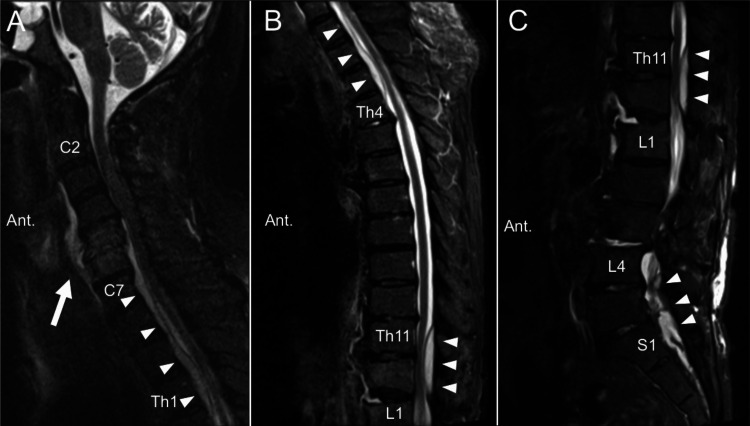
Preoperative radiological images. Sagittal whole-spine T2-weighted magnetic resonance images showing a retropharyngeal abscess (white arrow) and epidural abscess (white arrowheads). Hyperintensity of C5 and C6 vertebral bodies suggest pyogenic spondylitis. Fluid collection in the intervertebral spaces of C5/6, Th12/L1, L3/4, L4/5, and L5/S1 appear to be spondylodiscitis (A: cervicothoracic regions, B: thoracic regions, C: thoracic-lumbar-sacral regions).

These findings were suggestive of epidural abscess, spondylodiscitis, and pyogenic spondylitis. A retropharyngeal abscess was concurrently diagnosed. Pyogenic spondylosis appeared to have resulted from a retropharyngeal abscess. Cervical spondylosis and adult spinal deformity were also identified (Figure 1).

**Figure 2:**
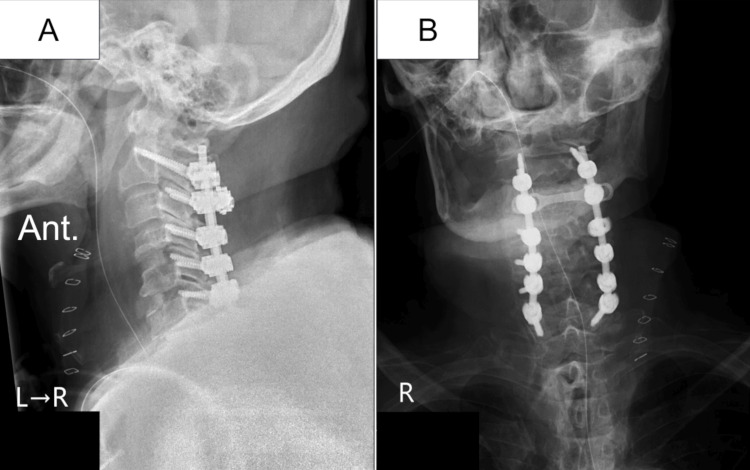
Radiographic images after the first operation. Anterior cervical discectomy and fusion of C4/5 and C5/6 and posterior cervical fixation from C2 to C7. The bone graft at C5/6 extruded anteriorly, however, postoperative dysphagia did not occur (A: lateral projection; B: anterior-posterior projection).

The patient was then referred to neurosurgery. The daily antibiotic management regimen was changed to meropenem (1.0 g twice daily) and vancomycin (1.0 g twice daily). In addition to this management, on day 8, we performed anterior cervical discectomy, fusion of the C4/5 and C5/6 levels, and posterior fixation from C2 to C7 in a single session because we thought that the cervical lesion could have caused pain in the upper and lower extremities. Abscess at the intervertebral level of C5/6 was simultaneously drained, the infectious intervertebral discs were debrided, and an autologous iliac bone graft was placed in the intervertebral spaces of C4/5 and C5/6 (Figure 2) [7]. We chose the anterior approach to drain the abscess, debride, and stabilize the infectious intervertebral lesions and performed also the posterior approach to release the cervical spinal cord compressed by the cervical spondylosis and the formation of an abscess.

**Figure 3:**
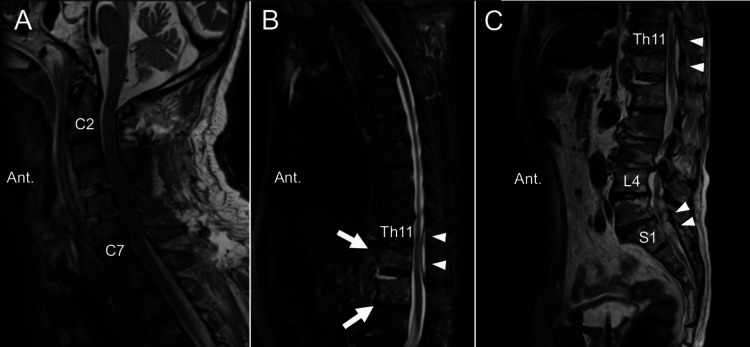
Magnetic resonance images after circumferential cervical fixation. Cervical epidural abscesses, pyogenic spondylitis, and spondylodiscitis resolved after cervical fixation surgery. The persistent epidural abscess at the lower thoracic and lumbosacral levels is identified (white arrowheads). Hyperintensity in the vertebral bodies of Th12 and L1 signifies de novo pyogenic spondylitis due to spread of infection (white arrows)(A: cervicothoracic regions; B: thoracic regions; C: thoracic-lumbar-sacral regions).

Antibiotic treatment was continued for a week after the first operation, but the lower back pain increased. Another follow-up whole-spine MRI examination showed resolution of the cervical pyogenic spondylitis and epidural abscess; however, persistent epidural abscess and pyogenic spondylitis were suspected below the Th12 level (Figure 3).

**Figure 4:**
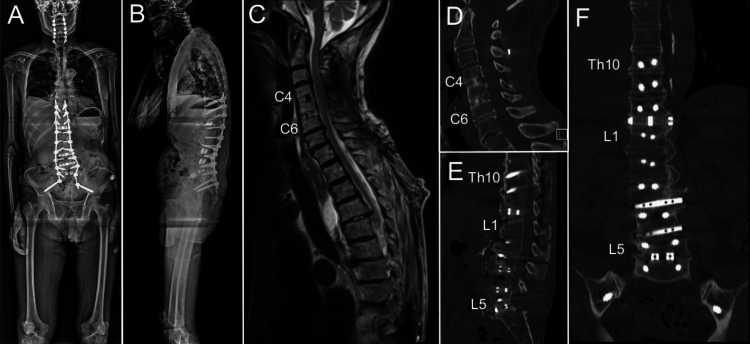
Radiographic images after the second operation Oblique and posterior lumbar intervertebral fixations concurrently performed with posterior long fusion from Th10 to the ilium. The Cobb angle and sagittal vertical axis were 15.9° and 50.5mm, respectively (A: lateral projection; B: anteroposterior projection). Sagittal spinal magnetic resonance imaging five months after the second surgery reveals resolution of the retropharyngeal abscess, epidural abscess, spondylodiscitis, and pyogenic spondylitis (C). Achievement of bone fusion is identified on computed tomography images six months after the second surgery (D-F)

On day 18, we performed OLIF at Th12/L1, L3/4, and L4/5, PLIF at L5/S1, and posterior fixation from Th10 to the ilium via PPSs (Figures 4A, 4B). OLIF and PLIF were performed to debride the infectious intervertebral discs, drainage abscess, and acquire stabilization. As adult spinal deformity coexisted, we thought that thoracic-lumbar-iliac posterior fusion was necessary. PPSs were selected to avoid invasiveness related to this operation. After the second surgery, teriparatide was initiated to facilitate bone fusion. Antibiotic treatment was terminated eight days later after confirming the decrease in inflammation (white blood cell count: 7560/μL, and C-reactive protein: 1.75 mg/dL) (Table 1). The patient underwent rehabilitation therapy and gradually recovered from it. Two months after the second operation, the patient was discharged temporarily. However, as the patient still required support in the activities of daily living, he and his family wished for the continuation of rehabilitation therapy. Therefore, the patient was readmitted and discharged again 116 days after the second surgery. The patient reported slight numbness of the fingertips bilaterally. One month after discharge, the patient presented to our outpatient clinic, ambulating independently. The Japan Orthopaedic Association cervical myelopathy evaluation questionnaire score was 13/17. At the time of this report, no apparent recurrence of the epidural abscess or pyogenic spondylitis was observed (Figure 4C). Bone fusion following fixation surgery was observed on follow-up images six months after the second surgery (Figures 4D-4F).

**Table 1:** Chronological change in blood sample examination. The inflammation decreases gradually after transvenous antibiotic treatment and two-staged surgery.

	On admission	Three days after admission	Eight days after the second surgery
White blood cell (normal range: 3500-9700/μl)	8840/μl	14250/μl	7560/μl
C-reactive protein (normal range: 0.00-0.30mg/dl)	32.15 mg/dl	22.45 mg/dl	1.75 mg/dl

## Discussion

Here, we describe a case of pyogenic spondylitis concurrently diagnosed with epidural abscess and spondylodiscitis successfully managed with circumferential cervical and thoracic-lumbar-sacral fixation in two sessions. This study is the first to report successful management of pyogenic spondylosis spreading from the cervical to the sacral region using this surgical strategy. In the first session, anterior cervical discectomy and fusion and posterior cervical fixation were performed to drain abscess, debride the infected intervertebral discs, decompress the cervical spinal cord, and obtain stabilization. Subsequently, OLIF, PLIF, and long-level posterior fixation from Th10 to the ilium via the PPS were performed for the thoracic-lumbar-sacral lesions in the second session. In the second operation also, the drainage of abscess, debridement of the infected discs, and spinal stabilization were aimed. Cervical spondylosis and adult spinal deformity were simultaneously resolved. 

Pyogenic spondylitis can be diagnosed based on clinical symptoms, radiological examinations, and laboratory tests. The disease manifests as fever, nausea, vomiting, local pain, weight loss, lethargy, impaired motion of infection, and neurological deficits [4]. However, the diagnosis can be delayed in cases where typical symptoms are not apparent or when other differential diagnoses are more prominent [4]. In our case, pleurisy on the initial computed tomography images was suspected to be the cause, and neurological symptoms were considered to be manifestations related to cervical spondylosis. Although antibiotic treatment had already been administered, pyogenic spondylosis possibly resulted from the retropharyngeal abscess spreading from the cervical to the sacral levels.

Pyogenic spondylosis can be managed surgically if conservative treatments fail. The conventional surgical approach for pyogenic spondylosis involves debridement of the anterior spinal components at the site of infection [7]. Typically, an iliac autograft and an anterior, posterior, or combined approach are considered for stabilizing the vertebral bodies or correcting kyphotic changes [7]. However, due to the invasiveness of surgery and the variety of infectious sites, surgical strategies can vary in clinical settings.

In our case, we performed surgery as we believed that cervical pyogenic spondylosis and spondylodiscitis caused the aggravated pain in the upper and lower extremities. As pyogenic spondylitis and spondylodiscitis did not recur and the epidural abscess resolved as seen on the MRI during follow-up, this strategy was useful in resolving the cervical and upper thoracic lesions.

For thoracic-lumbar-sacral lesions, we performed a second operation. MRI after the first surgery revealed that the lesions were resistant to antibiotic therapy alone. Pyogenic spondylodiscitis affected the Th12/L1, L3/L4, and L4/L5 levels, at which debridement and fixation were performed using OLIF. Although the efficacy of oblique intervertebral lumbar fusion has been previously reported [10,11], this method has been applied only to single-level lesions. Our case is unique in that we performed OLIF for multi-level lesions. PLIF was performed in addition to OLIF, and posterior long-level fixation from Th10 to the ilium via the PPS was performed. Minimally invasive surgery using a PPS for pyogenic spondylitis and spondylodiscitis has been described [8,12,13], which was also effective in our case.

As a limitation of this study, this is a single case of pyogenic spondylitis successfully managed with this method. Our patient had cervical spondylosis and adult spinal deformity. Therefore, circumferential cervical fixation and OLIF for multi-level lesions could have been beneficial also in resolving those coexistent diseases. The efficacy of our surgical strategy should be evaluated in similar cases in further research.

## Conclusions

As described in our case, cervical circumferential fixation, OLIF, and PLIF with long posterior thoracic-lumbar-sacral fixation via the PPS may be useful for resolving pyogenic spondylosis. The replacement of intervertebral discs with autologous iliac crests or intervertebral spacers after debridement and stabilization with posterior fixation via minimally invasive surgery may successfully resolve pyogenic spondylitis. Because this is a single case report successfully treated with this surgical strategy, it should be discussed in further study whether our operation can be standardized for pyogenic spondylitis similar to our case.
